# From Disease Control to Disease Modification: The Atopic Dermatitis Disease Activity Index

**DOI:** 10.1111/all.70036

**Published:** 2025-08-29

**Authors:** Thomas Bieber, Laura Maintz, Ganesh E. Phad, Marie‐Charlotte Brüggen

**Affiliations:** ^1^ Christine Kühne‐Center for Allergy Research and Education Medicine Campus Davos Davos Switzerland; ^2^ Allergy Unit, Department of Dermatology University Hospital Zürich Switzerland; ^3^ Bieber Dermatology Consulting Feldafing Germany; ^4^ Center for Skin Diseases University Hospital of Bonn Bonn Germany

**Keywords:** atopic dermatitis, biomarkers, disease modification, drug development, remission

To the Editor,

The concept of disease modification (DM) is established in disorders such as rheumatoid arthritis [1] where the tissue damage is irreversible and the goal of DM lies in slowing down the disease progress. While the notion of DM in atopic dermatitis (AD) was introduced a decade ago [2], it has been elaborated more recently [3] as well as treat‐to‐target approaches [4]. Since irreversible tissue remodeling lacks in AD, it has been proposed that a full inflammation control might lead to a status of DM, that is, an off‐therapy long‐lasting deep remission. Most of the recently approved systemic therapies reach short and mid‐term high clinical responses as measured by endpoints such as the Investigator Global Assessment (vIGA) 0/1 or Eczema Area Severity Index (EASI)‐90 during drug exposure in a subgroup of patients. An off‐therapy long‐lasting clinical and subclinical deep remission represents a paradigm shift which requires to addressing a number of variables for successful drug development [5]. Here, we aim to support the ongoing discussion on the critical issue of the outcome parameters for DM by discussing the concept of disease activity and the value of the AD disease activity index (ADDAI).

A key aspect is reaching a state of deep remission corresponding to a full clinical remission as well as the lack of an objectively measurable biological disease activity, potentially leading to an off‐therapy long‐term deep remission (Figure 1). To capture deep remission as a possible outcome for clinical trials, the ADDAI has been suggested [3]. The ADDAI is a composite score addressing 3 dimensions: (i) clinician‐reported outcomes (ClinRO), (ii) a patient‐reported outcome (PRO), and (iii) a biomarker (BM) tracking the subclinical level of inflammation (Table 1). The clinical disease activity should be measured by validated instruments for primary endpoints. For obvious regulatory reasons, we suggest the use of both vIGA (requested by the US agency FDA) and EASI (favored by the European agency, EMA). For PRO, the Patient‐Oriented Eczema Measure (POEM), Peak pruritus‐NRS‐11 (PP‐NRS 11), Dermatology Life Quality Index (DLQI), Recap of atopic eczema (RECAP) or Atopic Dermatitis Control Tool (ADCT) may be considered. To monitor DM, they should be assessed regularly during drug exposure time (see time X on Figure 1) and during an off‐therapy follow‐up for at least 12 months to cover the previously suggested time frames for long‐term remission (see time Y on Figure 1). However, the frequent assessment of multiple PRO may be cumbersome for many patients. Thus, we suggest to pragmatically consider the periodical assessment with the PP‐NRS‐11 as a short validated PRO with strong correlation to clinical severity, itch, pain, and most other PROs; for children, the respective adapted and validated scales, the worst itch scale for patients aged 6–11 years and the care‐giver‐reported worst scratch/itch NRS for children aged 6 months to 5 years (Table 1). Mobile applications and wearable devices might be helpful to integrate the PRO in patients' lives. We suggest an initial assessment of PRO weekly, of ClinRO and BM every 3 months at the regular clinical visits to comprehensively monitor DM without taking much additional time and effort. The intervals might be prolonged once reaching the absence of signs and symptoms of AD.

**FIGURE 1:**
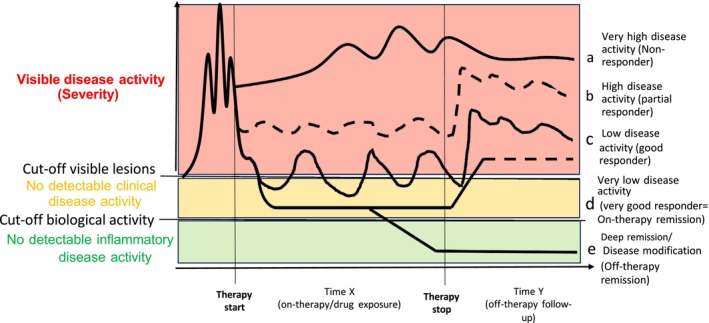
Examples of different scenarios of disease activity and response to therapy. (a) Very high disease activity: lack of significant improvement despite adequate management. (b) High clinical disease activity: despite adequate management with combined systemic and local therapies, stopping the systemic therapy leads to new exacerbations. (c) Low clinical disease activity: the control of the disease is reached with combined systemic and local therapies. Stopping the systemic therapy leads to loss of control and new exacerbation. (d) Very low clinical disease activity (“on‐therapy remission”): full control of AD solely under monotherapy. Stopping the systemic therapy leads to loss of control and new exacerbation. (e) Deep remission (“off‐therapy remission”): a very low clinical disease activity and lack of detectable biological disease activity as measured by BM. This state would allow stopping the therapy followed by an observational period (time Y) after which the status of DM could be confirmed.

**TABLE 1:** The ADDAI is suggested to monitor the disease activity of AD and achievement of deep remission as a composite score consisting of (i) Clinician‐reported outcomes ClinRO: Validated Investigator Global Assessment (vIGA) and Eczema Area Severity Index (EASI); (ii): Patient‐Reported Outcomes (PRO: Peak pruritus‐NRS) (PP‐NRS; worst itch scale for children aged 6–11 years and the care‐giver reported worst scratch/itch numeric rating scale for children aged 6 months to 5 years), if applicable Patient‐Oriented Eczema Measure (POEM); Dermatology Life Quality Index (DLQI), Recap of atopic eczema (RECAP) or Atopic Dermatitis Control tool (ADCT) with (iii) biomarker(s) yet to be defined and validated. The ADDAI should be assessed before, on‐ and off‐therapy with an ADDAI of 0–1 indicating remission and a potential timepoint to stop systemic therapy.

Remission outcomes	ClinRO	PRO	Biomarker
Deep Long‐term remission: ADDI = 0–1/achievement of target outcomes for ≥ 12 months	vIGA 0	PP‐ NRS 0 or 1 Worst itch scale 0–1 (6–11 years) Worst scratch/itch NRS 0–1 (0.5–5 years)	Yet to be defined and validated under consideration of distinct endotypes depending on ethnicity, age and other factors
	EASI 0	POEM 0–2: (almost) clear	
		DLQI 0 or 1: no impairment of Qol	
		RECAP or ADCT	

Assessment of ADDAI	ClinRO	PRO	Biomarker
Before therapy	X	X	X
On‐therapy assessment period ≥ 6 months^a^	X (every 3 months)	X (weekly)	X (every 3 months)
Therapy stop if ADDAI = 0–1	X	X	X
Off‐therapy follow‐up for ≥ 12 months	X (every 3 months)	X (weekly for 3 months, in case of remission afterwards once/every 2 weeks or monthly)	X (every 3 months)

Atopic Dermatitis Disease Activity Index (ADDAI)		
Clinican‐reported outcome	Patient‐reported outcome	Biomarker *(tape‐stripping/serum)*
vIGA	PP‐NRS 11 or POEM	Yet to be defined and validated
EASI	DLQI	
	RECAP or ADCT	

*Note:* The values for the different endpoints are indicative and need confirmation in a consensual approach.

^a^ In case of extremely high disease activity despite appropriate treatment, the diagnosis of AD (exclusion of differential diagnoses: Diagnostic criteria for AD, skin biopsy), patients' compliance and choice of patient‐individual current therapy should be reconsidered.

The definition of appropriate BM in the ADDAI remains the most challenging aspect in the quantitative evaluation of biological disease activity. The candidate BM should appropriately monitor the subclinical inflammatory activity in the skin with a predictive value for the off‐therapy deep remission. Different approaches may be chosen for BM identification, including invasive (e.g., serum markers) or noninvasive (e.g., swabbing, tape stripping) ones. Since repetitive measurements may be warranted to monitor remission, a noninvasive approach may be favorable, especially for the pediatric population. Type 2 inflammation‐associated BM, such as TARC/CCL17 and/or measuring the status of tissue memory of inflammation (or a proxy BM thereof) are conceivable candidates. In any case, BM will require validation and regulatory qualification for their numerical implementation.

The state of deep remission as defined by the ADDAI could mimic the “natural DM” observed upon the spontaneous remission before puberty in more than 50% of childhood AD. A deep dive into the mechanisms underlying this natural DM will provide crucial information to discover new pharmacological targets and innovative therapies to reach DM.

## Author Contributions

Idea, concept and writing of the manuscript: T.B. Design of the Figure 1: T.B. Design of Table 1: L.M. Critical reading and commenting of the manuscript: L.M., G.E.P., and M.‐C.B.

## Disclosure

T.B. was speaker and/or consultant and/or Investigator for AbbVie, Affibody, Almirall, Amagma/Triveni, AnaptysBio, AOBiom, Anergis, Apogee, Arena, Aristea, Artax, Asana Biosciences, ASLAN pharma, Astria, Attovia, BambusTx, Bayer Health, Belenos, BioVerSys, Böhringer‐Ingelheim, Bristol‐Myers Squibb, BYOME Labs, Cantargia, CellDex, Connect Pharma, Daichi‐Sanyko, Dermavant, DICE Therapeutics, DirigentBio, Domain Therapeutics, DS Pharma, EQRx, EMD Serono, Galderma, Galapagos, Gilead, Glenmark, GSK, Incyte, Innovaderm, Janssen, Kirin, Kymab, LEO, LG Chem, Lilly, L'Oréal, Mabylon, MSD, Medac, Micreos, Nektar, Nextech, Novartis, Numab, OM‐Pharma, Ornavi, Overtone, Pfizer, Pierre Fabre, Protagonist Tx, Q32bio, RAPT, Samsung Bioepis, Sanofi/Regeneron, Scienta Lab, TIRmed, UCB, Union Therapeutics, UPStream Bio, YUHAN. He is founder and chairman of the board of the nonprofit biotech “Davos Biosciences AG” within the international Kühne‐Foundation and founder of the consulting firm “Bieber Dermatology Consulting”. L.M. has served as an investigator for AbbVie, Anaptys Bio, Almirall, Amgen, Bioprojet, Bristol‐Myers Squibb, Eli Lilly, Galderma, LEO Pharma, Numab, OM Pharma, Pfizer, Sanofi/Regeneron, UCB; an advisor for AbbVie, Almirall, LEO Pharma, Sanofi/Regeneron; received speaker honoraria from AbbVie, Almirall, Eli Lilly, LEO Pharma, Sanofi/Regeneron; and received research funding from CK‐CARE, Eli Lilly, LEO Pharma, and Sanofi/Regeneron. G.E.P. has no disclosures. M.‐C.B. reports grants (not for the current project) from LEO Foundation, LEO Pharma, Swiss National Science Foundation, Innovation Pool, GSK, Astra Zeneca; speaker and/or consulting fees from Sanofi, Almirall, GSK, LEO Pharma, AbbVie.

## Conflicts of Interest

The authors declare no conflicts of interest.

## Data Availability

This is not relevant.
